# The double life of a chemotherapy drug: Immunomodulatory functions of gemcitabine in cancer

**DOI:** 10.1002/cam4.7287

**Published:** 2024-05-21

**Authors:** Alaina C. Larson, Kenadie R. Doty, Joyce C. Solheim

**Affiliations:** ^1^ Eppley Institute for Research in Cancer & Allied Diseases University of Nebraska Medical Center Omaha Nebraska USA; ^2^ Fred & Pamela Buffett Cancer Center University of Nebraska Medical Center Omaha Nebraska USA; ^3^ Department of Biochemistry & Molecular Biology University of Nebraska Medical Center Omaha Nebraska USA; ^4^ Department of Pathology, Microbiology, & Immunology University of Nebraska Medical Center Omaha Nebraska USA

**Keywords:** cancer biology, chemotherapy, immunology, pancreatic cancer

## Abstract

Although the development of immunotherapies has been revolutionary in the treatment of several cancers, many cancer types remain unresponsive to immune‐based treatment and are largely managed by chemotherapy drugs. However, chemotherapeutics are not infallible and are frequently rendered ineffective as resistance develops from prolonged exposure. Recent investigations have indicated that some chemotherapy drugs have additional functions beyond their normative cytotoxic capacity and are in fact immune‐modifying agents. Of the pharmaceuticals with identified immune‐editing properties, gemcitabine is well‐studied and of interest to clinicians and scientists alike. Gemcitabine is a chemotherapy drug approved for the treatment of multiple cancers, including breast, lung, pancreatic, and ovarian. Because of its broad applications, relatively low toxicity profile, and history as a favorable combinatory partner, there is promise in the recharacterization of gemcitabine in the context of the immune system. Such efforts may allow the identification of suitable immunotherapeutic combinations, wherein gemcitabine can be used as a priming agent to improve immunotherapy efficacy in traditionally insensitive cancers. This review looks to highlight documented immunomodulatory abilities of one of the most well‐known chemotherapy agents, gemcitabine, relating to its influence on cells and proteins of the immune system.

## 1 INTRODUCTION

The immune system is a network comprised of cells, proteins, tissues, and organs which function to protect the host from foreign pathogens and tumor development. Although the immune system has intrinsic anticancer properties, it is not infallible and notably liable for failure in the case of those individuals who succumb to cancer.^1^ The reality of this insufficiency prompted the development of therapies to reinvigorate immune reactivity, aptly called immunotherapies, and includes the likes of vaccines, cytokines, adoptive T‐cell transfer, and monoclonal antibodies (e.g., immune checkpoint inhibitors).^2^ The scope of these immunotherapies, much like the components of the immune system itself, is widespread and encompasses agents that both invoke and inhibit the immune response as modes of therapeutic management.^3^, ^4^, ^5^, ^6^ Immunotherapies, particularly immune checkpoint inhibitors, have revolutionized treatment and patient outcomes for several cancer types, such as Hodgkin's lymphoma, melanoma, non‐small cell lung cancer, and renal cancer.^7^, ^8^, ^9^, ^10^, ^11^, ^12^, ^13^, ^14^ Despite these promising developments, multiple other cancers have not displayed sensitivity to tested immunotherapies in the current setting. This unresponsive phenotype is often derived from insufficient mutational burden, inhibitory immune checkpoint expression patterns, and defects in antigen presentation.^15^, ^16^, ^17^, ^18^ Immunotherapy‐ineligible patients instead rely on transiently effective, resistance‐prone chemotherapy drugs as the backbone of disease management.^19^, ^20^, ^21^

As the need for improved treatment options intensifies, attention has turned to “old‐school” chemotherapies as an untapped source of potential. Although chemotherapeutics were initially approved for their cancer‐killing properties, some are now known to be potent immunomodulators, capable of suppressing or invigorating the immune system.^22^, ^23^, ^24^, ^25^, ^26^, ^27^ The repurposing of these old school drugs with their novel immune‐editing abilities may allow for improved antitumor outcomes in the form of combination therapies. Specifically, chemotherapeutic immunomodulators can be paired with immunotherapies, wherein the chemotherapy drug can act as a priming agent and allow for enhanced immunotherapeutic efficacy in cancers that are normally unresponsive. One such drug that exhibits both chemotherapeutic and immunomodulatory abilities is gemcitabine.

Gemcitabine is a chemotherapy drug initially approved by the Food and Drug Administration in 1996 for the treatment of locally advanced and metastatic pancreatic cancer,^28^, ^29^ and it remains active in clinical use today for the treatment of breast, non‐small cell lung, and ovarian cancer, in addition to pancreatic cancer.^30^ Gemcitabine's use as a monotherapy is infrequent, and it is most often prescribed in combination regimens with other chemotherapies, including platinum and taxol‐based agents.^31^, ^32^, ^33^, ^34^ Gemcitabine's reputation as a favorable combination partner is derived in part from its limited toxicity, with adverse effects like myelosuppression, hair loss, nausea, and vomiting reported as mild or rarely of clinical significance.^35^

**FIGURE 1:**
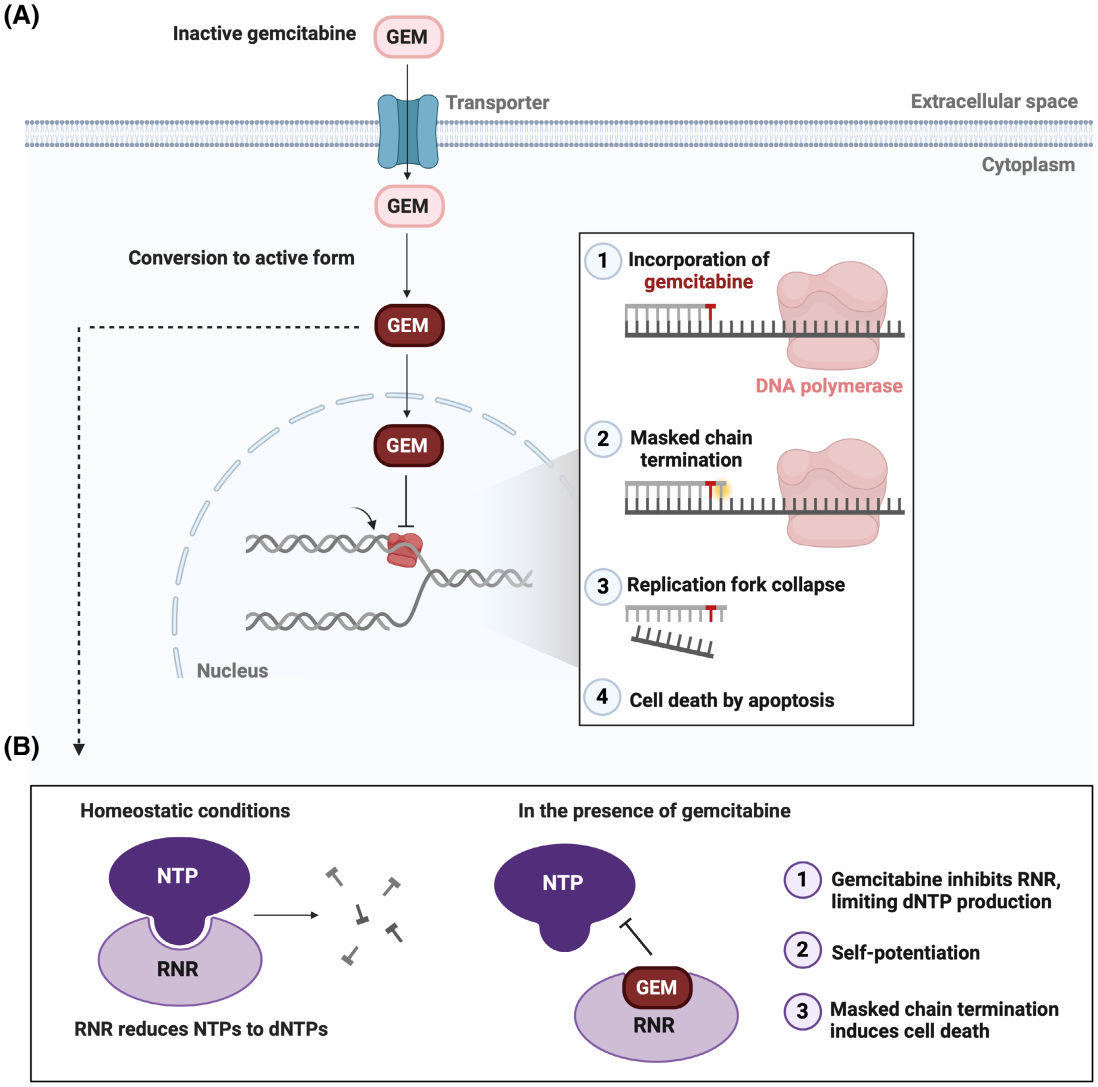
Gemcitabine's chemotherapeutic mechanisms of action. The figure depicts the primary modes by which gemcitabine induces cancer cell death (others exist which are not described). (A) Gemcitabine in its prodrug form enters the cell via a nucleoside transporter. In the cytoplasm, gemcitabine is converted to its nucleoside‐mimicking active state. DNA polymerase incorporates gemcitabine into the DNA chain and an additional nucleotide is added, securing gemcitabine in the DNA strand (masked chain termination). The polymerase is unable to proceed which promotes fork collapse and cell death. (B) Gemcitabine occupies the active site of RNR. RNR inhibition disrupts dNTP pools, increasing the likelihood of gemcitabine's incorporation into DNA (self‐potentiation), and initiating cell death by masked chain termination. dNTP, deoxyribonucleotide triphosphate; GEM, gemcitabine; NTP, ribonucleotide triphosphate; RNR, ribonucleotide reductase.

Gemcitabine belongs to the antimetabolite class of chemotherapeutics, joining pharmaceuticals like 5‐fluorouracil and methotrexate.^36^ Its cytotoxic function is exerted via masked chain termination in which gemcitabine, *masking* as a nucleoside (deoxycytidine), is mistakenly incorporated into the DNA strand. Following addition of a single nucleotide, DNA polymerase is released, the replication fork collapses, and the cell succumbs to apoptosis (Figure 1A).^37^, ^38^ The fork collapse is accompanied by ataxia telangiectasia mutated and Rad3‐related (ATR) pathway activation, and it has been demonstrated that inhibition of ATR interferes with PD‐L1 upregulation that would otherwise occur via interferon regulatory factor 1 (IRF1) signaling.^39^, ^40^ In a concurrent mechanism, gemcitabine covalently binds to the active site of ribonucleotide reductase (RNR), an enzyme responsible for conversion of ribonucleotides (NTPs) to deoxyribonucleotides (dNTPs). Impairment of RNR function disrupts dNTP levels, thereby improving gemcitabine's propensity for DNA incorporation in a process called self‐potentiation (Figure 1B).^38^

**FIGURE 2:**
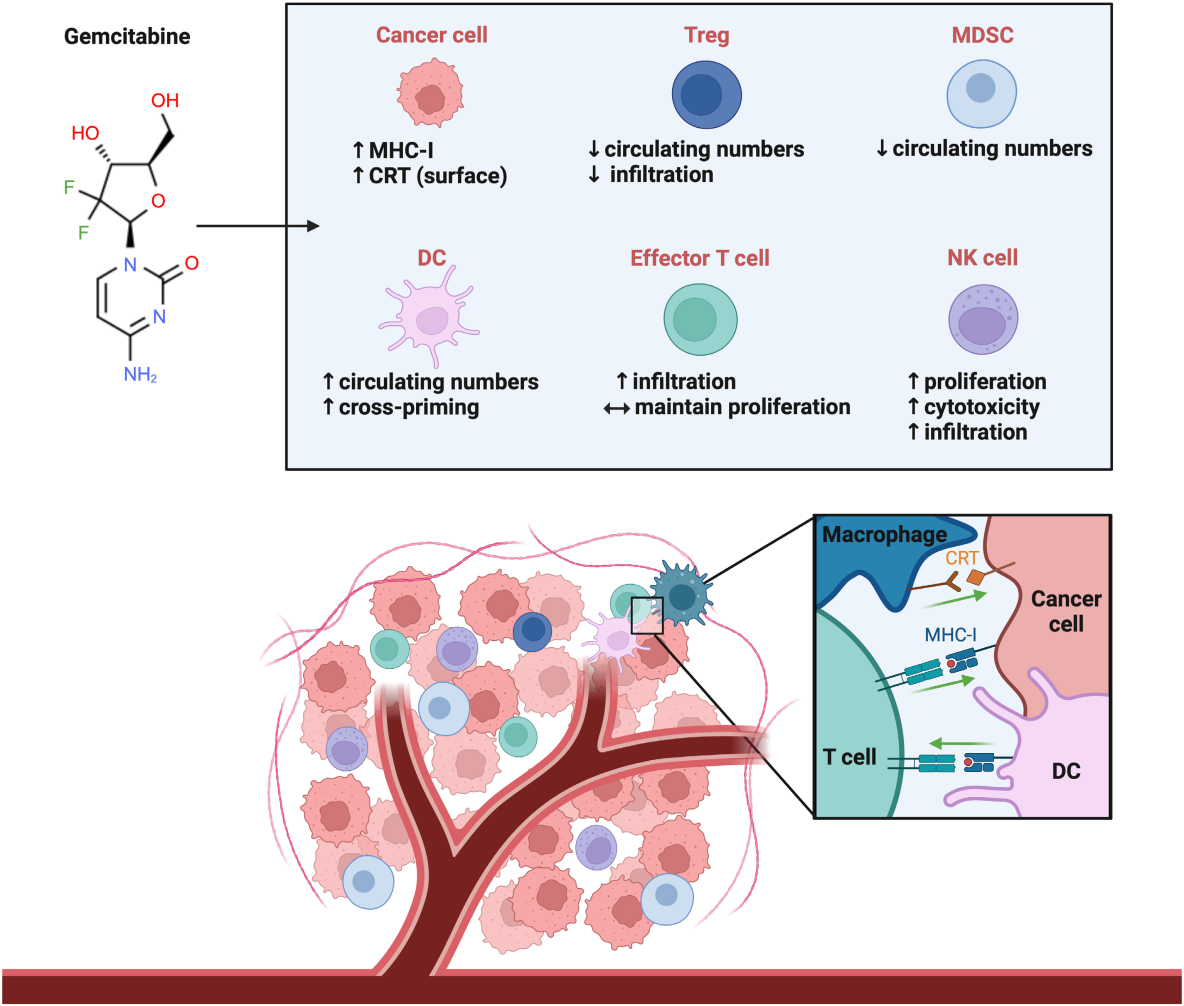
Pro‐immune effects of gemcitabine in cancer. The figure highlights gemcitabine's positive immunomodulatory properties on immune cells and immune‐related proteins reported in at least one type of cancer. Gemcitabine reduced levels of circulating (MDSCs, Tregs) and intratumoral (Tregs) immunosuppressive cell populations in various cancer models. In addition, gemcitabine intensified the antitumor abilities of NK cells, including their tumor infiltration and lytic activity. The proliferative potential of NK cells was also improved by gemcitabine in patients with malignant mesothelioma. In pancreatic cancer patients treated with gemcitabine, the number of DCs in circulation increased. Gemcitabine also improved the cross‐priming of CD8^+^ T cells in tumor‐bearing mice. The proliferative capacity of effector T cells was not diminished after gemcitabine treatment in pancreatic cancer patients, and tumor infiltration of CD8^+^ and CD4^+^ effector T cells was stimulated by gemcitabine in a mouse model of ovarian cancer. Gemcitabine augmented expression of immune‐promoting proteins, including surface CRT on cancer cells. Plasma membrane‐resident CRT can act as an “eat me” signal and induce engulfment by phagocytic cells. Increased expression of the MHC‐I complex, a cytotoxic T cell‐activating molecule, has also been documented in cancer models following gemcitabine exposure. [Green arrows indicate signal activation.] CRT, calreticulin; DC, dendritic cell; MDSC, myeloid‐derived suppressor cell; MHC‐I, major histocompatibility complex class I; NK, natural killer cell; Treg, regulatory T cell.

Multiple studies have indicated that gemcitabine holds not only cytotoxic capabilities but also boasts additional immune‐modifying functions. In fact, gemcitabine treatment has demonstrated immuno‐altering properties across several cancer types. For example, gemcitabine increases expression of immune system proteins like MHC class I chain‐related protein A and B (MICA/B), major histocompatibility class I (MHC‐I), programmed death‐ligand 1 and 2 (PD‐L1, PD‐L2), calreticulin (CRT), and others.^24^, ^41^, ^42^ The immunomodulatory abilities of gemcitabine extend beyond alterations of tumor cells themselves—this drug also influences both the behaviors and relative abundance of several immune cell populations (Figure 2).^43^, ^44^, ^45^ Because of its potential to improve patient sensitivity to immunotherapy‐mediated intervention, gemcitabine's immune‐editing capacity relative to immune cells and tumor cells will be highlighted within this review.

## 2 IMMUNOMODULATORY EFFECTS OF GEMCITABINE ON IMMUNE CELLS

Tumor cells are not the sole perpetrators of malignant behaviors; rather, they require and extort neighboring immune cells to further their proliferative and migratory objectives. Thus, the presence and abundance of certain tumor‐promoting immune cells within the microenvironment can have consequences for disease progression.^46^ Finding mechanisms either to deplete such immunosuppressive cells or to increase the presence of antitumor immune cells is an ongoing area of investigation.^46^ Pharmaceutical intervention may hold the answer to such an immunological dilemma and is described in the context of gemcitabine in the section below.

### 2.1 Regulatory T cells

Regulatory T cells, or as they are commonly denoted, Tregs, play an important role in regulation of the immune response via release of suppressive cytokines and inhibition of T cell proliferation.^47^, ^48^, ^49^ Normally, these immunosuppressive functions are critical for the prevention of autoimmunity, promotion of self‐tolerance, and maintenance of homeostasis.^50^ However, while such immunoregulation is protective in a healthy host system, it can become pathological in the context of cancer. Consequently, Tregs' infiltration is often characterized as a blockage to effective tumor immunity and thus a poor prognostic factor in several solid tumor cancers.^51^, ^52^, ^53^ Identifying mechanisms to facilitate the depletion or inactivation of Tregs may allow for cancer management by restoring the antitumor immune response.

Gemcitabine exposure is known to influence Tregs across several models. A modest but significant increase in survival was illustrated in an orthotopic pancreatic cancer mouse model, which demonstrated reduced levels of Tregs following gemcitabine treatment.^23^ The increase in survival appeared to be in part derived from the depletion of Tregs, as the administered gemcitabine dosage was suboptimal and did not affect the volume of the primary tumor.^23^ In peripheral blood samples from mesothelioma patients, gemcitabine exposure was associated with decreased Treg proliferation.^54^ In conjunction, a study conducted by Eriksson et al. observed a higher percentage of circulating Tregs in chemonaïve pancreatic cancer patients in comparison with healthy donors.^44^ However, treatment with gemcitabine reduced the average percent of peripheral blood Tregs in these patients.^44^ In addition, the mean effector: Treg ratio of this patient cohort was augmented during the gemcitabine cycle, suggesting that gemcitabine likely increased the favorable effector T cell population, lessened the number of circulating, suppressive Tregs, or expedited an advantageous combination of both.^44^

### 2.2 Myeloid‐derived suppressor cells, monocytes, and macrophages

Like Tregs, myeloid‐derived suppressor cells (MDSCs) are well known for their immune‐quelching activities.^55^ Both immature cells of granulocytic and monocytic lineage are classified as MDSCs and exert their immunosuppressive functions primarily through inhibition of T‐cell activation, but also by mitigating macrophage‐mediated cytokine secretion and NK cell cytotoxicity.^56^, ^57^, ^58^ The role of MDSCs in maintaining homeostatic conditions remains somewhat ambiguous, albeit these cells are known to promote favorable immunosuppression in semi‐allogeneic states of pregnancy and transplantation.^59^, ^60^ Under cancerous conditions, the immunosuppressive functions of MDSCs are exploited by tumors in attempts to avoid immune detection and maintain their proliferative agenda. Circulating MDSC levels are often enhanced in several cancer types, including melanoma, pancreatic cancer, and squamous cell carcinoma of the head and neck.^61^, ^62^, ^63^

Interestingly, gemcitabine appears to reduce MDSC levels. In a study conducted by Le et al., mice were subcutaneously injected in the flank with murine mammary tumor cells.^64^ Because MDSCs can accumulate in peripheral lymphoid organs, the population of MDSCs in the spleen was evaluated after 3 weeks.^64^ Upon harvesting, MDSCs had amassed within the spleen and accounted for approximately 30% of splenic cells in the untreated group.^64^ However, MDSCs in the spleen were depleted to 10% in mice that received weekly gemcitabine treatments.^64^ The findings of this study were similar to those of another group which showed that gemcitabine decreased the MDSC population localized within the spleen in mice bearing large mesothelioma or lung tumors (flank, subcutaneously inoculated).^65^ Despite the gemcitabine‐induced MDSC loss, there was no corresponding decrease in effector CD4^+^ or CD8^+^ T cells within the spleens of these animals.^65^ Gemcitabine treatment also affects MDSC populations within human patients. Eriksson et al. showed that gemcitabine administration reduced peripheral MDSCs in patients with pancreatic cancer to levels near that of the healthy donor cohort.^44^ Similar results were published in a study of individuals diagnosed with recurrent breast cancer who were prescribed gemcitabine.^66^ Both prior and post‐chemotherapy administration, the percentage of circulating MDSCs was evaluated.^66^ Levels of MDSCs in the peripheral blood significantly decreased following gemcitabine treatment.^66^ Furthermore, the percentage of circulating MDSCs after the gemcitabine cycle was not statistically different from that of the healthy volunteer group, indicating gemcitabine favorably modulates MDSC levels in refractory breast cancer cases.^66^

Monocytes, and upon their differentiation, macrophages, play a critical part in mediating inflammation, namely through phagocytosis, secretion of cytokines, and activation of the adaptative immune response.^67^ Phenotypically distinct subtypes of monocytes and macrophages have unique roles in the suppression or promotion of tumor development.^68^ In patients with advanced pancreatic cancer, Soeda et al. reported an increase in the absolute number and percentage of circulating CD14^+^ monocytes after gemcitabine treatment.^69^ However, because additional phenotypic markers were not used to further stratify these monocytes (e.g., HLA‐DR^low/neg^),^68^ it is difficult to infer the clinical significance. Gemcitabine's pro‐tumor effect extends to macrophages, as this chemotherapy drug has been observed to facilitate intratumoral infiltration by anti‐inflammatory M2 macrophages.^70^ Mice with orthotopic xenograft pancreatic tumors were found to have more macrophages within the tumors if the mice had been treated with gemcitabine, and conditioned culture media from gemcitabine‐exposed pancreatic cancer cells induced polarization of macrophages to the M2 phenotype.^70^ In a mouse model of breast cancer, gemcitabine treatment stimulated monocyte development, and subsequently increased the presence of CCR2^+^ monocytes and macrophages in the lungs, thereby potentiating tumor metastasis.^71^

Neutrophils are often credited as the initial responders in sites of injury, inflammation, or infection.^72^ In cancer, the pro‐tumor and antitumor effects of neutrophils remain inconclusive.^72^ Gemcitabine monotherapy, as well as its combination with other chemotherapies, may induce levels of neutropenia (and thrombocytopenia) that can be of clinical concern.^73^, ^74^, ^75^ Surprisingly, incidence of chemotherapy‐induced neutropenia (CIN) has been correlated with improved survival in cancer patients,^76^ and for gemcitabine‐treated pancreatic cancer patients, early‐onset CIN was identified as a predictor of more favorable prognosis.^77^

### 2.3 Natural killer cells

Natural killer cells, or NK cells as they are commonly called, are a population of lymphoid cells which were discovered in the 1960s.^78^ As indicated by their name, this cell type is *naturally* cytotoxic, requiring no previous antigen exposure to induce lysis of a target cell.^78^, ^79^, ^80^ NK cell‐mediated lysis and granule secretion are primarily regulated through the presence and/or absence of various receptors and molecules on the surface of target cells.^81^, ^82^, ^83^, ^84^, ^85^ This cytolytic capacity is critical to the host's defense against cells that have been either virally infected or malignantly transformed.^86^ The presence of NK cell populations, which have corresponding antitumor abilities, is considered to be a favorable phenotype in an array of solid tumors.^87^

Notably, gemcitabine has demonstrated an ability to influence NK cell populations in cancer . Gürlevik et al. established an R0 resectable transgenic mouse model of pancreatic cancer in which mice developed a single, pancreas‐specific tumor.^45^ Following R0 resection, gemcitabine was administered in a localized fashion to the pancreas remnants.^45^ Mice that received adjuvant gemcitabine exhibited enhanced infiltration of NK cells at the resection margins in comparison to the untreated control group.^45^ Furthermore, this gemcitabine‐perpetuated increase in NK cells was therapeutically relevant as depletion of NK cells enhanced local disease recurrence in this cohort.^45^ Gemcitabine also stimulated NK cell‐mediated cytotoxicity in a study conducted by Zhang et al. Splenic NK cells were purified from subcutaneous tumor‐burdened mice and cocultured in vitro with lung tumor cells.^43^ The NK cells obtained from the gemcitabine‐treated mice lysed a higher percentage of tumor cells than those collected from the untreated mice, suggesting that gemcitabine positively enhances NK cell activity.^43^ Gemcitabine also appears to modulate the proliferative capacity of circulating NK cells in individuals with malignant mesothelioma.^54^ Mesothelioma patients received first‐line platinum‐pemetrexed chemotherapy, and those without progressive disease were later given either gemcitabine as maintenance therapy or basic supportive care (BSC) treatment.^54^ NK cell proliferation was significantly upregulated in the cohort which received maintenance gemcitabine treatment in comparison to the BSC group.^54^

### 2.4 Cytotoxic and helper alpha beta T cells, gamma delta T cells, dendritic cells, and B cells

The function of the adaptive immune system is carried out by the activities of several cell populations, including T cells, dendritic cells (DCs), and B cells. Alpha beta T cells and gamma delta T cells are distinguished based on their expression of the alpha beta T cell receptor or gamma delta T cell receptor, respectively.^88^ Within the alpha beta designation, CD8^+^ cytotoxic T cells initiate direct cell‐mediated lysis upon recognition of MHC‐I‐bound antigens, while CD4^+^ helper T cells release pro‐inflammatory cytokines after identification of MHC‐II‐displayed antigens.^1^ DCs and B cells are both professional antigen‐presenting cells and promote activation of T cells, though B cells are mainly distinguished for their secretion of antibodies.^1^

The importance of T cells to the therapeutic efficacy of gemcitabine was made evident by the discovery that this drug is ineffective against tumors in athymic nude mice.^89^ Gemcitabine contributes to cross‐priming of DCs (by inducing tumor cell apoptosis and other mechanisms), and thereby causes antigen‐specific stimulation of CD8^+^ T cells.^90^, ^91^ Furthermore, gemcitabine has been observed to increase the number of DCs in peripheral blood samples from pancreatic cancer patients.^69^

Plate et al. evaluated the kinetics of gemcitabine's impact on immune cell populations and found that initial drops in T and B cell numbers were reversed as treatment of pancreatic cancer patients progressed.^92^ Anti‐CD3 antibody stimulation of peripheral blood mononuclear cells obtained from gemcitabine‐treated pancreatic cancer patients showed no diminution of T cell proliferation capability in comparison to controls.^44^, ^93^ In a mouse model, gemcitabine caused some reduction of CD4^+^ and CD8^+^ T cell numbers, but a more than double impairment of B cell proliferation.^94^ This gemcitabine‐mitigated B cell activity was accompanied by a substantial reduction of the antibody response to a model tumor antigen that was expressed by mesothelioma tumor cells.^94^

Ovarian tumor‐bearing mice that received gemcitabine had more CD4^+^ and CD8^+^ T cells infiltrating the tumors.^95^ Gemcitabine has been noted to have positive effects on CD8^+^ cytotoxic T cell antitumor activity, possibly as a consequence of diminishing the viability of MDSCs in the tumor microenvironment,^65^ as well as by its abovementioned ability to enhance T cell priming. In the context of mesothelioma, gemcitabine treatment of patients led to increased expression of co‐stimulatory molecules by CD4^+^ helper T cells and CD8^+^ cytotoxic T cells, indicating phenotypic changes in these cell populations.^54^

In addition to the alpha beta T cell receptor‐expressing CD4^+^ and CD8^+^ T cells, the gamma‐delta T cell receptor‐expressing subset can also have antitumor activities. However, gamma delta T cell‐mediated cytotoxicity is independent of antigen presentation by MHC molecules.^96^ Shimizu et al. showed that low‐dose gemcitabine pretreatment augmented the cytolytic activity of zoledronic acid‐stimulated gamma delta T cells against urinary bladder cancer cells, and this combination also reduced tumor burden in vivo.^97^ In a study of patients treated with gemcitabine alone or with both gemcitabine and gamma delta T cell therapy, no statistically significant survival differences were found.^98^ However, there was a correlation between the persistence of gamma delta T cells in the blood and the absence of disease recurrence, suggesting that a clinical trial with a larger enrollment of patients would possibly yield more evidence of efficacy for this combination therapy.^98^

## 3 IMMUNOMODULATORY EFFECTS OF GEMCITABINE ON CANCER CELLS

Although off‐target effects certainly exist, the intended quarries of chemotherapeutics (gemcitabine included) are the cancer cells themselves.^99^, ^100^ As such, it is not surprising that a number of the immunomodulatory effects of gemcitabine manifest within the tumor. Tumor cells employ multiple tactics to evade immune surveillance, for example, the downregulation and upregulation of proteins that promote and curtail the immune response, respectively.^101^, ^102^, ^103^ Thus, pharmaceutical‐induced expression of proteins that can restore the immune response is an active area of interest. Gemcitabine's impact on several of these immune‐related proteins is described below.

### 3.1 Major histocompatibility complex class I

Major histocompatibility complex class I (MHC‐I) is a molecule that is expressed at the surface of all nucleated cells and has a critical function in the presentation of abnormal peptides to CD8^+^ cytotoxic T cells.^104^ Recognition of these peptides as atypical (e.g., virus‐ or tumor‐derived) induces T cell‐mediated lysis against the infected or malignant cell.^105^ Therefore, MHC‐I is a vital component in instigating an immune response under pathological conditions. As such, many tumors downregulate expression of MHC‐I as a means of immune escape.^106^, ^107^, ^108^, ^109^, ^110^ Thus, finding ways to fully restore surface expression of this molecule could play a major role in reinvigorating the T cell‐mediated antitumor immune response. Gemcitabine has demonstrated a propensity to increase expression of MHC‐I in several human cancer cell lines, including pancreatic, colon, breast, lung, and cholangiocarcinoma.^24^, ^111^, ^112^, ^113^, ^114^ Our own work in human pancreatic cancer cell lines demonstrated that gemcitabine increased MHC‐I mRNA and protein levels, as well as cell surface expression and stability.^115^ Of note, we also observed that gemcitabine modified MHC‐I‐displayed peptides on a pancreatic cancer cell line and improved these peptides' predicted affinity and immunogenicity.^115^ Liu et al. and Principe et al. showed that gemcitabine's stimulation of MHC‐I protein expression was recapitulated in murine models of lung cancer and pancreatic cancer, respectively.^24^, ^114^ In vivo analysis revealed that while gemcitabine alone could enhance MHC‐I expression in murine pancreatic tumors, it was not sufficient to promote effector T‐cell infiltration. However, with the triple combination of gemcitabine, an immune checkpoint inhibitor, and a TGF‐β‐signaling inhibitor, T cell penetration into the tumors was not only restored, but the mice also exhibited increased overall survival.^24^ These experiments strengthen the potential clinical relevance of gemcitabine's immunomodulatory capacity and indicate its ability to act successfully in combination therapies.

### 3.2 Calreticulin

Calreticulin (CRT) is a calcium‐binding, sarcoplasmic reticulum (SR) and endoplasmic reticulum (ER)‐resident chaperone protein.^116^, ^117^ This protein harnesses many functions and is involved in regulation of calcium homeostasis, intracellular signaling, gene expression, and assistance in protein folding.^118^, ^119^, ^120^ Under physically or chemically induced cellular stress, CRT can be translocated from the ER to the cell surface.^121^, ^122^ Following its incorporation into the plasma membrane, CRT acts as a damage‐associated molecular response (DAMP) or “eat‐me” signal at the surface of the cell, initiating phagocytic‐mediated engulfment.^123^ Elevated expression of surface CRT is correlated with improved clinical outcomes and considered a favorable prognostic factor in acute myeloid leukemia, ovarian cancer, and non‐small cell lung cancer.^124^, ^125^, ^126^ Thus, the translocation of CRT is a desired contributor to the antitumor immune response, and of interest in this review, potentially achievable through gemcitabine exposure. In vitro analysis of murine bladder and pancreatic cancer cell lines revealed gemcitabine's capacity to increase surface expression of CRT.^41^ Such results were mirrored in human cholangiocarcinoma and lung cancer cells, as well as a murine model of lung cancer.^43^, ^127^, ^128^ Interestingly, Smith et al. observed that human pancreatic cancer cells cultured in medium with gemcitabine not only had increased surface expression of CRT but also a higher frequency of engulfment via monocyte‐derived dendritic cells, though it cannot be stated that this was specifically due to CRT upregulation.^113^ Nonetheless, because membrane‐bound CRT is a potent DAMP and inducer of immune cell‐mediated death, combining gemcitabine and other immune‐enhancing therapies could lead to strategies to increase cancer‐directed phagocytosis.

### 3.3 PD‐L1

Programmed death‐ligand 1 (PD‐L1) was the first discovered ligand of the immune cell‐expressed programmed cell death protein 1 (PD‐1).^129^ The interaction of PD‐1 and PD‐L1 promotes the inactivation of immune cells, thereby decreasing their proliferation and capacity for cytokine production.^130^ PD‐L1 is categorized as an *immune checkpoint*, and it is critical in the regulation of self‐tolerance and homeostasis. However, this checkpoint has a nefarious connotation in the setting of cancer, perpetuating T cell impairment and subduing immune‐mediated responses against malignant cells.^131^, ^132^ Expression of PD‐L1 is significantly increased in many solid tumors, including nasopharyngeal carcinoma as well as bladder, breast, and gastric cancers.^133^, ^134^, ^135^ PD‐L1 upregulation is characterized as a poor prognostic factor and affiliated with lower survival rates in the aforementioned cancers.^133^, ^134^, ^135^ However, PD‐L1 overexpression on tumor cells can also be advantageous in terms of enhancing the efficacy of monoclonal antibody therapies targeting this protein. Immune checkpoint inhibitors, such as anti‐PD‐L1 therapeutics, have been clinically successful and paramount in the restoration of antitumor T cell responses.^136^, ^137^ Thus, paradoxical as it may seem, the inadvertent upregulation of PD‐L1 by pharmaceuticals could be beneficial in conversion of a traditionally insensitive cancer to one that is immunotherapeutically targetable. Gemcitabine has been observed to increase PD‐L1 across several models of pancreatic cancer, including established cell lines, primary cell line‐derived xenografts, murine cell lines, and a mouse model of pancreatic cancer.^24^, ^138^, ^139^ For ovarian cancer cell lines, gemcitabine stimulated mRNA levels and surface expression of PD‐L1 by threefold and sixfold, respectively.^95^ Furthermore, a study by Jung et al. observed that gemcitabine enhanced expression of PD‐L1 by a human colorectal cancer cell line and significantly improved tumor infiltration and binding of an anti‐PD‐L1 antibody in vivo.^140^ Therefore, gemcitabine‐stimulated PD‐L1 expression may promote advantageous responses in the clinic via immune checkpoint combinations.

## 4 POTENTIAL MECHANISMS

The propensity for gemcitabine (and other chemotherapeutics with similar activities) to engage in both cytotoxic and immunomodulatory behavior remains an active area of exploration. With such an array of immuno‐modifications induced by gemcitabine, it is unlikely that a singular channel is responsible. Instead, this section will highlight several potential mechanisms by which gemcitabine may influence the immunophenotype of cells.

Gemcitabine's seemingly selective reduction of immunosuppressive cell populations may be in part due to the proliferative nature of these cells. Both MDSCs and Tregs have the potential for rapid expansion,^141^, ^142^, ^143^ and thus may be more prone to gemcitabine incorporation than other immune populations. Suzuki et al., showed that gemcitabine increased apoptosis of MDSCs (Gr‐1^+^/CD11b^+^), but did not affect non‐suppressive lymphocyte populations in vitro.^65^ In a similar vein, intratumoral Treg populations underwent higher rates of cellular division compared to conventional T cell populations in a pancreatic cancer model, and thus their reduction by gemcitabine was more likely due to preferential proliferative‐targeting by this drug.^23^

Transcriptional regulation is often shared between the immune system's “on switch” (e.g., MHC‐I) and the corresponding “off switch” (e.g., PD‐L1) to prevent extended and unintentional reactivity beyond the initial immune response. For example, interferon‐sensitive response elements (ISREs) located in the promoters of the PD‐L1 gene and MHC‐I‐associated genes confer their sensitivity to interferon‐mediated signaling.^144^, ^145^ Likewise, nuclear factor kappa‐light‐chain‐enhancer of activated B cells (NF‐κB)binding sites make these genes' susceptible to NF‐κB regulation (e.g., via TNFα signaling).^144^, ^146^ In a murine model of pancreatic cancer, gemcitabine upregulated secretion of several cytokines, including interferon gamma (IFNγ) and tumor necrosis factor alpha (TNFα),^24^ both of which are known transcriptional inducers of MHC‐I and PD‐L1.^144^, ^146^ Thus, it is plausible that in vivo gemcitabine instigates cytokine‐mediated signaling to modify expression of immune‐related proteins.

This ability for gemcitabine to stimulate secretion of inflammatory cytokines may be a byproduct of its inherent chemotherapeutic mechanisms (i.e., DNA damage and nucleotide pool disruption). In nasopharyngeal carcinoma, MHC‐I induction by gemcitabine and a gemcitabine/cisplatin combination was found to be dependent on the STING type I interferon‐dependent pathway,^147^ suggesting that chemotherapy‐induced DNA fragmentation triggers inflammatory cytokine production. Nucleotide depletion by gemcitabine has also been shown to stimulate expression of several interferon‐stimulated genes (ISGs).^148^ Activity of these ISGs was inhibited by reintroduction of certain nucleotides with gemcitabine co‐treatment, indicating that it is the nucleotide inhibition by gemcitabine which invokes expression of these ISGs.^148^ Stimulation of immune‐associated genes via pharmaceutical‐induced disruption of nucleotide pools has been previously observed and could be a concomitant effect of the infection mimicry state induced by these drugs.^149^, ^150^, ^151^, ^152^

The signaling events which relay nucleotide loss to these genes requires additional investigation. Of note, a recent report by Mullens et al., revealed that stimulation of MHC‐I associated genes by a nucleotide‐disrupting drug was abrogated through inhibition of positive transcription elongation factor b (P‐TEFb), a protein necessitated for efficient transcription.^153^ Thus, it appears that drug‐induced nucleotide depletion is dependent on the activity of P‐TEFb, but discerning whether this is a conserved mechanism among agents with similar modes of action (e.g., gemcitabine) will require further exploration.

## 5 CONCLUSIONS AND PERSPECTIVES

Chemotherapy has proven to be a front‐line method of disease management for most cancer types. However, many forms of cancer suffer from high refractory rates or chemoresistance, and there is a continued need to identify courses of action to improve prognosis beyond the canonical neoadjuvant and adjuvant therapies.^154^ Such a reality has prompted a reinvestigation into the mechanisms of standard chemotherapy drugs and brought forth additional immune‐editing abilities for several of these anticancer drugs.^155^ It is believed that the immunostimulatory properties of chemotherapy drugs, such as gemcitabine, can be used to enhance the efficacy of immunotherapies against cancers that are traditionally insensitive. Gemcitabine exerts favorable impacts on both immune cell populations and tumor cells, including depletion of Tregs and MDSCs, infiltration of NK cells and effector T cells, as well as invigoration of immune‐stimulating surface CRT and MHC‐I expression (Figure 2). Within the last 3 years, more than 15 clinical trials have evaluated immunotherapy combination strategies which include gemcitabine (Table 1).^156^, ^157^, ^158^, ^159^, ^160^, ^161^, ^162^, ^163^, ^164^, ^165^, ^166^, ^167^, ^168^, ^169^, ^170^, ^171^ Although the results varied between trial settings, cancer types, and patient cohorts, positive outcomes were documented within the published trials. For example, the combination of gemcitabine/nab‐paclitaxel and an immune checkpoint inhibitor (anti‐PD‐1) improved 1‐year survival from historical averages in patients with metastatic pancreatic cancer.^158^ Thus, there is continued interest in investigating multi‐therapy approaches and identifying the cancer types and patient populations most responsive to these combinations.

**TABLE 1:** Completed clinical trials testing gemcitabine and immunotherapy combinations.^a^

Phase and no. of patients enrolled	Cancer	Chemotherapy/SOC	Immunotherapy/target	Primary outcome	Identifier no.	Reference
Phase II—128 patients	Biliary tract cancer (advanced)	Gemcitabine/cisplatin	Durvalumab (anti‐PDL1) Tremelimumab (anti‐CTLA‐4) Durvalumab/tremelimumab	The ORR from the chemotherapy +durvalumab was 72%, from chemotherapy+durvalumab+trem‐limumab it was 70%, and it was 50% for those who received chemotherapy then chemotherapy + durvalumab+tremlimumab.	NCT03875235	^156^
Phase II—38 patients	Biliary tract cancer (advanced)	Gemcitabine/oxaliplatin	Camrelizumab (anti‐PD‐1)	Combination improved PFS by 2.6 months when compared to historical averages. OS was also increased by the combination therapy (extended by 3.3 months). Combination therapy was a tolerable regimen.	NCT03486678	^157^
Phase II—105 patients	Pancreatic (metastatic)	Gemcitabine/nab‐paclitaxel	Nivolumab (anti‐PD‐1) Sotigalimab (anti‐CD40, agonist) Nivolumab and sotigalimab	Combination of nivolumab + chemotherapy extended 1‐year OS (57.7%) from the historical average (35%), *n* = 34). Other combinations did not improve 1‐year OS compared to historical numbers.	NCT03214250	^158^
Phase II—180 patients	Pancreatic (metastatic)	Gemcitabine/nab‐paclitaxel	Durvalumab/ tremelimumab (anti‐PD‐L1/anti‐CTLA‐4)	Combination therapy did not improve survival.	NCT02879318	^159^
Phase II—68 patients	Biliary tract cancer (advanced)	Gemcitabine/cisplatin	Nivolumab (anti‐PD‐1)	Combination had similar PFS (6 months, 59.4%) as standard chemotherapy treatment. However, OS was improved by combination therapy (2 years, 35.4%) when compared to historical averages (15%–22%), indicating there is a subset of patients who may benefit.	NCT03101566	^160^
Phase III—184 patients	Large B cell lymphoma (refractory or early relapsed)	R‐DHAP/R‐ICE/R‐GDP	Lisocabtagene maraleucel (autologous, CD19‐directed CAR T cell therapy)	Inclusion of CAR T cell therapy increased median event‐free survival (10.1 months) in comparison to the standard of care group (2.3 months).	NCT03575351	^161^
Phase Ib—21 patients	Pancreatic (advanced)	Gemcitabine	Ipilimumab (anti‐CTLA‐4)	Combination therapy was a tolerable regimen.	NCT01473940	^162^
Phase I—42 patients	Pancreatic (advanced, treatment refractory)	Gemcitabine	Defactinib/pembrolizumab (FAK inhibitor/anti‐PD‐1)	Combination therapy was a tolerable regimen.	NCT02546531	163, 164
Phase Ib—23 patients	Head and neck squamous cell carcinoma (advanced)	Gemcitabine/cisplatin	Toripalimab (anti‐PD‐1)	Combination therapy was a tolerable regimen.	NCT04947241	^165^
Phase II—32 patients	Biliary tract cancer (metastatic or unresectable)	Gemcitabine/cisplatin	Nivolumab (anti‐PD‐1)	Of the patients who were evaluable, the ORR was 55.6%, with 18.6% obtaining a CR.	NCT03311789	^166^
Phase III—303 patients	Pancreatic (advanced)	Gemcitabine/nab‐paclitaxel	Algenpantucel‐L (vaccine; allogeneic pancreatic cancer cells engineered to express murine α[1,3]GT)	Median OS (14.9 months) was not improved from that of the standard of care group (14.3 months).	NCT01836432	^167^
Phase II—27 patients	Primary mediastinal B cell lymphoma (relapsed/refractory)	Gemcitabine/vinorelbine/PLD	Camrelizumab (anti‐PD‐1)	Combination therapy was tolerable. The ORR of the combination therapy was 74%, with 56% achieving CR. Historical numbers not provided; disease is rare.	NCT03346642	^168^
Phase I/II—32 patients	Pancreatic (resected)	Gemcitabine (adjuvant)	TG01GM‐CSF (vaccine; 7 RAS peptides representing the 7 most common KRAS codon 12 and 13 oncogenic mutations/granulocyte macrophage colony‐stimulating factor)	Combination therapy increased median OS (33.3months) compared to historical data (17.1–26.5 months) for adjuvant gemcitabine, which was the SOC at the time. TG01/GM‐CSF is safe to use in conjunction with chemotherapy. A positive immune response (defined as a DTH response and/or a positive T cell proliferation assay) was observed for >90% of patients.	NCT02261714	^169^
Phase Ib—20 patients	Squamous non‐small cell lung cancer (advanced)	Gemcitabine/cisplatin	Sintilimab (anti‐PD‐1)	Combination therapy was a tolerable regimen.	NCT02937116	^170^
Phase II—71 patients	Ovarian cancer (relapsed, but platinum and gemcitabine‐sensitive)	Gemcitabine/carboplatin	DCVAC/OvCa (vaccine; autologous DCs pulsed with human OV‐90 and SK‐OV‐3 ovarian cancer cells pretreated with high hydrostatic pressure for the induction of immunogenic cell death)	Combination therapy was a tolerable regimen but did not extend median PFS compared to chemotherapy alone arm (11.3 months vs. 9.5 months, respectively). However, it did improve median OS by 13.4 months).	NCT02107950	^171^

Abbreviations: α[1,3]GT, alpha‐1,3‐galactosyltransferase; CAR, chimeric antigen receptor; CD19, cluster of differentiation 19; CD40, cluster of differentiation 40; CR, complete response; CTLA‐4, cytotoxic T‐lymphocyte‐associated antigen 4; DCs, dendritic cells; DTH, delayed type hypersensitivity response; GM‐CSF, granulocyte macrophage colony‐stimulating factor; KRAS, Kirsten rat sarcoma viral oncogene homolog; FAK, focal adhesion kinase; PD‐1, programmed cell death protein 1; PD‐L1, programmed death‐ligand 1; PFS, progression‐free survival; PLD, pegylated liposomal doxorubicin; ORR, overall response rate; OS, overall survival; RAS, Rat sarcoma; R‐DHAP, rituximab/dexamethasone/cytarabine/cisplatin; R‐GDP, rituximab/gemcitabine/dexamethasone/cisplatin; R‐ICE, rituximab/ifosfamide/carboplatin/etoposide; SOC, standard of care.

^a^ Only published results with documented National Clinical Trial (NCT) identifier numbers from January 2020 to May 2023 are included. Combination studies conducted prior to 2020 or that are currently ongoing were excluded from the table. Active clinical trials can be found at ClinicalTrials.gov.

Although this review has focused mostly on the positive immune‐promoting effects of gemcitabine, it is important to note that some of its modulatory properties could be classified as suppressive and detrimental. The neutropenia and thrombocytopenia induced in cancer patients by gemcitabine can potentially lead to mortality, yet they are also statistically associated with improved survival.^172^, ^173^ Despite gemcitabine's propensity to reduce MDSCs in vivo,^65^ it may also activate the inflammasome pathway in these cells and accelerate tumor expansion through release of inflammatory cytokines.^174^ As noted above, gemcitabine has also been implicated in increasing tumor infiltration by M2‐polarized macrophages, a phenotype that is typically regarded as tumor‐promoting.^70^ In addition, as mentioned earlier in this review, gemcitabine treatment is correlated with increased production of monocytes, which by homing to sites such as the lungs and differentiating to immunosuppressive macrophages, can assist metastasis.^71^

Subsequently, further exploration must be conducted to accurately estimate gemcitabine's beneficial modulation of immune parameters from potential disadvantageous effects. Various investigations have sought biomarker correlates of gemcitabine treatment and patient outcomes,^175^, ^176^ and some studies have included hematological and immunological factors.^173^, ^177^ For example, Blomstrand et al. profiled blood proteins and cells in pancreatic cancer patients and evaluated whether any were prognostic for prolonged survival.^173^ The occurrence of thrombocytopenia was a positive prognostic marker for progression‐free survival, but myelosuppression had neither positive nor negative linkage to survival in this setting.^173^ Another example of a potential immune‐related biomarker is endoplasmic reticulum aminopeptidase 2 (ERAP2), which correlates with worse prognosis in pancreatic cancer patients and its expression in pancreatic cancer cells is increased by gemcitabine treatment.^177^ ERAP2 is a protein involved in the processing of peptide ligands for binding to MHC‐I,^178^ but it also has a role in the PI3K/AKT/mTOR pathway, and whether its immunological function and/or its cell signaling function is the rationale for its correlation with poor prognosis is not well understood. Bauer et al. showed that gemcitabine therapy decreased DC vaccine impact on B cell and CD8^+^ T cell responses, but the combination of the DC vaccine and gemcitabine still was clinically beneficial,^179^ showing the difficulty of establishing specific immunological biomarkers that correlate with overall clinical prowess. In the future, more in‐depth knowledge of the immunological events occurring after gemcitabine treatment, and their association with outcomes, may not only influence selection of immunotherapy partners, but also aid in the establishment of dosages and administration scheduling to achieve optimal responses and mitigate potential suppressive effects in the clinical setting.

## AUTHOR CONTRIBUTIONS

**Alaina C. Larson:** Conceptualization (lead); formal analysis (lead); funding acquisition (equal); project administration (equal); writing – original draft (lead); writing – review and editing (equal). **Joyce C. Solheim:** Funding acquisition (equal); project administration (equal); supervision (lead); writing – review and editing (equal). **Kenadie R. Doty:** Writing – review and editing (equal).

## FUNDING INFORMATION

This work was supported by the Fred & Pamela Buffett Cancer Center's Cancer Center Core Grant (P30CA036727), the Cancer Biology Training Program Grant (T32CA009476), and through the American Association of Immunologists Careers in Immunology Fellowship Program for Computational Scientists and Immunologists.

## CONFLICT OF INTEREST STATEMENT

The authors declare no conflict of interest.

## Data Availability

Data sharing is not applicable to this article as no new data were generated for this review.
